# Restriction of Posterior Tibial Translation During the Posterior Drawer Test in Internal or External Rotation Is Dependent on Peripheral Stabilizers of the Knee: A Biomechanical Robotic Investigation

**DOI:** 10.1177/03635465251317209

**Published:** 2025-02-19

**Authors:** Adrian Deichsel, Christian Peez, Wenke Liu, Michael J. Raschke, Alina Albert, Thorben Briese, Elmar Herbst, Christoph Kittl

**Affiliations:** † Department of Trauma, Hand and Reconstructive Surgery, University Hospital Münster, Münster, Germany; Investigation performed at the Department of Trauma, Hand and Reconstructive Surgery, University Hospital Münster, Münster, Germany

**Keywords:** knee, posterior cruciate ligament, posterior drawer test, peripheral stabilizers, rotational drawer

## Abstract

**Background:**

The posteromedial and posterolateral structures of the knee have previously been shown to be secondary restraints to posterior tibial translation (PTT). The effect of these structures may increase when performing the posterior drawer test in internal or external rotation.

**Purpose/Hypothesis:**

The purpose was to investigate the influence of the posteromedial and posterolateral structures on restricting PTT in neutral, external, and internal rotation. It was hypothesized that the posteromedial structures restrict PTT in internal rotation, while the posterolateral structures restrict PTT in external rotation.

**Study Design:**

Controlled laboratory study.

**Methods:**

A sequential cutting study was performed on 24 fresh-frozen human knee specimens utilizing a 6 degrees of freedom robotic test setup. After determining the native knee kinematics from 0° to 90° of knee flexion, an 89-N posterior drawer test in neutral, internal, and external rotation was performed at 0°, 30°, 60°, and 90° of knee flexion. In 8 knees, a motion-controlled protocol was applied, replicating the native motion while the force was measured. The reduction of the restraining force represented the percentage contribution of each cut. In 16 knees, a force-controlled protocol was applied, determining the increase in PTT after each cut. After calculating the native knee kinematics, the posterior cruciate ligament (PCL) was cut, followed by randomized sectioning of the posteromedial (medial collateral ligament, posterior oblique ligament) and posterolateral (lateral collateral ligament, popliteus complex) structures. Mixed linear models with the post hoc Dunn test were used for statistical analysis.

**Results:**

During motion-controlled testing, performing the posterior drawer test in internal or external rotation significantly decreased the contribution of the PCL in restraining PTT. The PCL was the primary restraint to PTT during the posterior drawer test in neutral rotation at all flexion angles (24.4%-61.2% contribution). The primary restraint to PTT during the posterior drawer test in internal rotation was the posterior oblique ligament at 0° (24.2% ± 14.1%), the medial collateral ligament at 30° (33.6% ± 11.4%), and the PCL at 60° and 90° (46.2%-57.8%). In external rotation, the primary restraint was the lateral collateral ligament at 0° (24.7% ± 10.5%) and the popliteus complex at 30° to 90° (56.4%-65.2%). During force-controlled testing, PTT in the PCL-deficient knee was significantly decreased when performing the posterior drawer test in internal or external rotation. Insufficiency of the posterolateral or posteromedial structures, in addition to insufficiency of the PCL, during the posterior drawer test in neutral rotation led to an additional significant increase in PTT of up to 7.6 mm (95% confidence interval [CI], 3.6-11.7). Insufficiency of the posterolateral structures led to a significant further increase in PTT during the posterior drawer test in external rotation of up to 12.6 mm (95% CI, 3.5-21.8). Insufficiency of the posteromedial structures led to a significant additional increase in PTT during the posterior drawer test in internal rotation of up to 14.9 mm (95% CI, 8.2-21.6).

**Conclusion:**

The peripheral ligamentous structures of the knee acted as secondary restraints to PTT in neutral rotation and became primary stabilizers in internal or external rotation, depending on the flexion angle.

**Clinical Relevance:**

This study may guide the clinician in diagnosing deficiencies of the posteromedial or posterolateral structures of the knee. In cases of isolated PCL deficiencies, PTT is reduced when the posterior drawer test is performed in internal or external rotation. However, an additional deficiency of the posteromedial or posterolateral structures further increases PTT.

Ruptures of the posterior cruciate ligament (PCL) frequently occur in combination with injuries of the posterolateral or posteromedial structures.^7,24,37,43^ The posterolateral complex is composed of the lateral collateral ligament (LCL) and arcuate complex, consisting of the popliteus tendon (PT), popliteofibular ligament (PFL), and auxiliary structures.^14,21^ The posteromedial complex is composed of the medial collateral ligament (MCL), the posterior oblique ligament (POL), the posteromedial capsule, and the semimembranosus tendon.^20,47^ It is generally accepted that the PCL is the primary restraint to posterior tibial translation (PTT).^4,16^ However, both the posterolateral and posteromedial complexes have previously been shown to restrict PTT when injured in isolation or in combination with the PCL.^6,9,11,13,36,48^ Based on anatomic and biomechanical observations, the posteromedial structures tighten in internal rotation and may therefore better restrict PTT in internal rotation, while the posterolateral structures tighten in external rotation and may therefore restrict PTT better in external rotation.^1,29^

Clinically, injuries of the PCL are typically evaluated by the use of the posterior drawer test.^29,40^ Additionally, the posterior drawer test in internal and external rotation was proposed as a clinical evaluation to differentiate between isolated PCL tears and combined posteromedial and posterolateral instability.^15,46,52^ However, the specific influence of the peripheral structures on restricting PTT in internal or external rotation is unclear thus far.

The purpose of this study was to investigate the influence of the peripheral ligamentous structures on limiting PTT during the posterior drawer test in neutral, external, and internal rotation. It was hypothesized that the posteromedial structures restrict PTT in internal rotation, while the posterolateral structures restrict PTT in external rotation.

## Methods

### Knee Specimen Preparation

A total of 24 unpaired cadaveric knee specimens (mean age, 66.5 ± 9.6 years) without previous knee surgery, high-grade osteoarthritis, or meniscal injuries were obtained from MedCure. The experiments were performed with permission from the institutional review board of the University of Münster (No. 2023-407-f-S). After each test, the knee was checked for ligamentous or meniscal injuries not related to the testing protocol.

Specimens were stored at −20°C and thawed for 24 hours at room temperature before preparation. The skin and subcutaneous fat were resected, leaving remaining soft tissue intact. The tibia and femur were secured in aluminum cylinders, 12 cm above and below the joint line, with 3-component polyurethane bone cement (RenCast; Gößl & Pfaff). The fibula was then cut 10 cm below the joint line and transfixed with a 3.5-mm cortical screw to the tibia.^39^ According to the descriptions of Merican et al,^27^ longitudinal transpatellar osteotomy was performed to allow visualization and subsequent cutting of the PCL attachment. No muscle loading was performed in this study. Specimens were wrapped in wet tissue paper to prevent drying.

### Robotic Test Setup

A validated setup consisting of a 6 degrees of freedom industrial robot (KR 60-3; KUKA) equipped with a force-torque sensor (F/T Theta; ATI Industrial Automation) was used for biomechanical testing in this study, as previously described.^2,5,30,45,49^ The robot allowed for position-controlled movements with an accuracy of ±0.06 mm as well as force-controlled movements with an accuracy of ±0.25 N and ±0.05 N·m. The test setup was optimized for the simulation of knee joint movements by simVITRO custom software (Cleveland Clinic BioRobotics Laboratory). A modified Grood and Suntay coordinate system was created for each specimen by digitizing landmarks on the femur and tibia with a tactile measuring arm (Absolute Arm 8320-7; Hexagon Metrology), which has an accuracy of ±0.05 mm.^22,47^ The sampling rate of the test setup was set to 500 Hz.

### Biomechanical Testing

Each specimen was flexed and extended 10 times to maximize hysteresis of each specimen.^26^ The neutral point of each knee was determined by manually minimizing all forces and torques acting on the knee in full extension. The passive path of the knee was then determined by flexing each knee from full extension to 90° of flexion while minimizing forces and torques in all axes, aside from the flexion-extension axis. An axial compression force of 50 N was applied to warrant contact between the femur and tibia during movements through the passive path.

**Figure 1.**
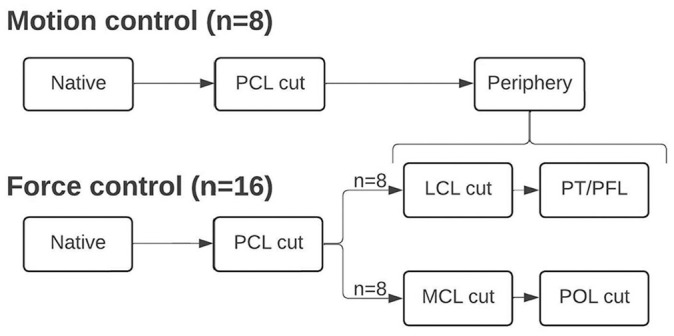
Cutting sequence during motion- and force-controlled testing.

A force-controlled testing protocol was applied, with displacement in response to given forces/torques recorded. At 0°, 30°, 60°, and 90° of flexion, the following was performed under axial compression of 200 N: 89-N anterior and posterior drawer forces as well as 89-N posterior drawer force with 5-N·m internal rotation torque and 5-N·m external rotation torque, simulating the posteromedial and posterolateral drawer tests (results presented in mm). In 16 specimens, the force-controlled testing protocol was repeated after each step to determine changes in PTT (in mm) for each cutting step (Figure 1). In 8 specimens, the kinematics of the native knee was transferred to a motion-controlled testing protocol (recording forces in response to given displacement). This protocol replicated the exact movements captured during testing of the native knee kinematics.^53^ The reduction of the force after the repetition of the native knee kinematics indicated the contribution of each subsequent cut in restricting PTT according to the principle of superposition.^4,10,40^ The structures with the highest contribution in each respective movement was characterized as the primary restraint.^31^ Structures with a contribution of ≥20% were defined as major restraints, and structures with a contribution of <20% were defined as minor restraints.

### Sequential Cutting

After determination of the native knee kinematics, the PCL was transected at its femoral insertion site via the previously created transpatellar approach. In case of a meniscofemoral ligament being present, it was transected, together with the PCL. After determining the PCL-deficient kinematics, the posteromedial and posterolateral complexes were cut with a scalpel in a randomized order. While cutting the posteromedial complex, the superficial MCL was resected, followed by the POL. While cutting the posterolateral complex, the LCL was cut, followed by the PT and PFL. During testing with the force-controlled protocol, in half of the specimens, either the posterolateral (n = 8) or posteromedial (n = 8) complex was cut, as cutting more than one side led to dislocation of the specimen, as observed during pilot testing.

### Statistical Analysis

Extraction of knee kinematics from the raw data of simVITRO was performed using MATLAB (Version R2024a; MathWorks). Statistical analysis was performed using Prism (Version 8; GraphPad Software). Mixed linear models with the Greenhouse-Geisser correction were used for all statistical analyses to assess the main effects and interactions of each independent variable (cutting state and flexion angle). The dependent variables were (1) PTT in neutral rotation, (2) PTT in external rotation, and (3) PTT in internal rotation. Pairwise comparisons were used to compare the contribution of the cutting states at different flexion angles. The results are presented as mean differences (MDs) with corresponding 95% confidence intervals (CIs). The post hoc Dunn test was performed to account for multiple testing. A *P* value of <.05 was deemed to identify significant differences.

A priori power analysis was performed using G*Power (Version 3.1).^8^ Based on means and standard deviations from previous studies^2,17,19,51^ on knee laxity, it was determined that a sample size of 8 would identify changes in translation and rotation of 2.0 mm and 2°, respectively, with 80% power, at a significance level of *P* < .05.

## Results

### Contribution of PCL in Restricting PTT (Motion Controlled)

**Figure 2.**
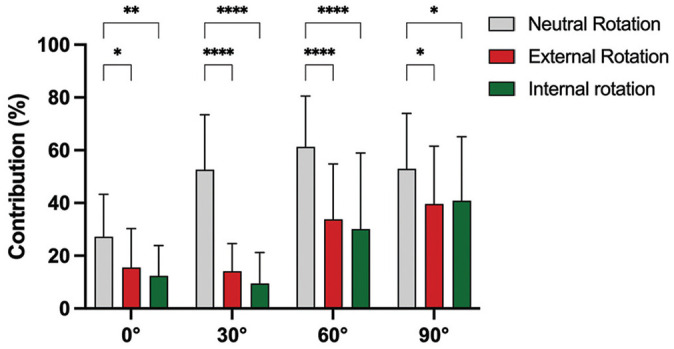
Contribution of the posterior cruciate ligament to restrict posterior tibial translation in neutral, external, and internal rotation. *P < .05; **P < .01; ***P < .001; ****P < .0001.

At all flexion angles, performing the posterior drawer test in either external or internal rotation significantly reduced the contribution of the PCL to restrict PTT compared with neutral rotation (*P* < .05) (Figure 2 and Appendix Table A1 [available in the online version of this article]).

### Influence of Rotation on PTT in PCL-Deficient Knee (Force Controlled)

**Figure 3.**
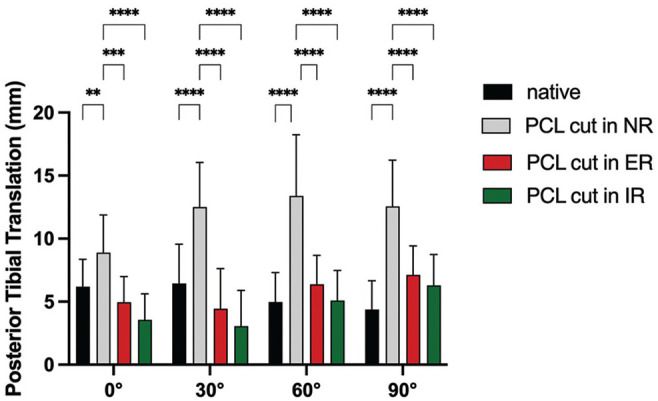
Effect of performing the posterior drawer test in neutral rotation (NR), external rotation (ER), and internal rotation (IR) on posterior tibial translation in the posterior cruciate ligament (PCL)-deficient knee. **P < .01; ***P < .001; ****P < .0001.

Cutting the PCL led to a significant increase in PTT at 0°, 30°, 60°, and 90° of flexion in comparison to the native state. Performing the posterior drawer test in either external or internal rotation significantly decreased PTT at all flexion angles (Figure 3) in comparison to neutral rotation (range of reduction of PTT, 3.9-9.5 mm).

### Contribution of Peripheral Structures in Restricting PTT (Motion Controlled)

**Figure 4.**
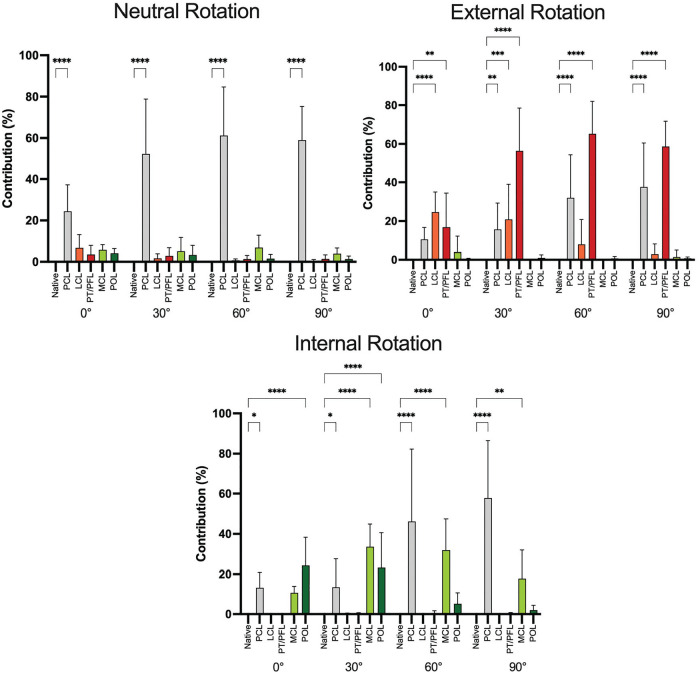
Contribution of the posterior cruciate ligament (PCL) and peripheral structures in restricting posterior tibial translation in neutral rotation, external rotation, and internal rotation. LCL, lateral collateral ligament; MCL, medial collateral ligament; PFL, popliteofibular ligament; POL, posterior oblique ligament; PT, popliteus tendon. *P < .05; **P < .01; ***P < .001; ****P < .0001.

In neutral rotation, the PCL was the only significant contributor to the restriction of PTT (Figure 4 and Appendix Table A1). In neutral rotation, the peripheral structures were only minor contributors to the restriction of PTT, without a significant contribution to restriction.

When performing the posterior drawer test in external rotation, the LCL was the primary restraint at 0° of flexion and a major restraint at 30° of flexion. The PT/PFL was a significant contributor to the restriction of PTT at all flexion angles. At 30°, 60°, and 90°, the PT/PFL was the primary restraint. The PCL was a significant contributor at 30°, 60°, and 90° of flexion. No significant contribution of the MCL or POL was found in external rotation at any flexion angle.

When performing the posterior drawer test in internal rotation, the POL was the primary restraint at 0° of flexion and a significant contributor at 30° of flexion. The MCL was found to be the primary restraint at 30° of flexion and a significant contributor at 60° and 90° of flexion. The PCL was found to be a significant contributor at all flexion angles and the primary restraint at 60° and 90° of flexion. No significant contribution of the LCL or PT/PFL was found in internal rotation.

### PTT in Neutral Rotation (Force Controlled)

**Figure 5.**
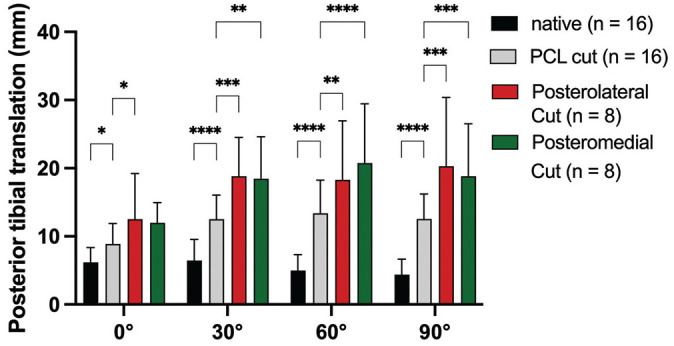
Posterior tibial translation after cutting the posterior cruciate ligament (PCL) and peripheral structures during the posterior drawer test in neutral rotation. LCL, lateral collateral ligament; MCL, medial collateral ligament; PFL, popliteofibular ligament; POL, posterior oblique ligament; PT, popliteus tendon. *P < .05; **P < .01; ***P < .001; ****P < .0001.

When performing the posterior drawer test in neutral rotation, cutting the PCL led to a significant increase in PTT at every flexion angle (Figures 3 and 5). Deficiency of the posterolateral structures led to significant additional instability at 0° (MD, 4.2 mm [95% CI, 0.4-8.1]; *P* = .03), 30° (MD, 7.3 mm [95% CI, 3.4-11.2]; *P* = .0003), 60° (MD, 5.5 mm [95% CI, 1.7-9.4]; *P* = .005), and 90° (MD, 7.6 mm [95% CI, 3.6-11.7]; *P* = .0003) in comparison to the PCL-deficient state. Deficiency of the posteromedial structures led to significant additional instability at 30° (MD, 3.0 mm [95% CI, 0.3-6.1]; *P* = .001), 60° (MD, 6.8 mm [95% CI, 3.6-10.0]; *P* ≤ .0001), and 90° (MD, 6.2 mm [95% CI, 3.0-9.5]; *P* = .0003) in comparison to the PCL-deficient state.

### PTT in External Rotation (Force Controlled)

**Figure 6.**
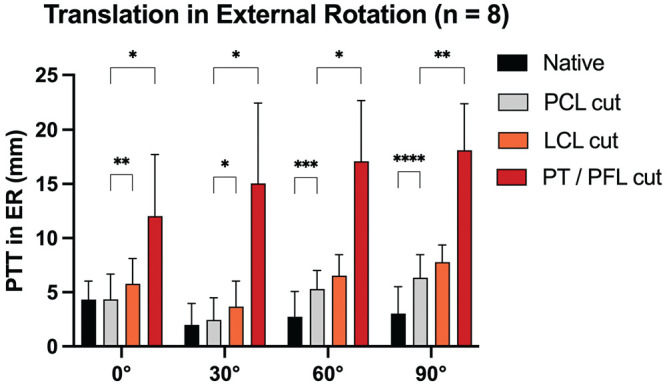
Posterior tibial translation after cutting the posterior cruciate ligament (PCL) and peripheral structures during the posterior drawer test in external rotation. LCL, lateral collateral ligament; PFL, popliteofibular ligament; PT, popliteus tendon. *P < .05; **P < .01; ***P < .001; ****P < .0001.

When performing the posterior drawer test in external rotation, cutting the PCL led to a significant increase in PTT at 60° (MD, 2.6 mm [95% CI, 1.6-3.5]; *P* = .0005) and 90° (MD, 3.3 mm [95% CI, 2.7-3.9]; *P* ≤ .0001) in comparison to the native state (Figure 6). Cutting the LCL led to a significant additional increase in PTT at 0° (MD, 1.4 mm [95% CI, 0.9-2.0]; *P* = .001) and 30° (MD, 1.2 mm [95% CI, 0.2-2.3]; *P* = .02) in comparison to the PCL-deficient state. Cutting the PT/PFL (after the LCL) led to an additional increase in PTT at 0° (MD, 7.7 mm [95% CI, 0.4-14.9]; *P* = .04), 30° (MD, 12.6 mm [95% CI, 3.5-21.8]; *P* = .02), 60° (MD, 11.8 mm [95% CI, 4.5-19.1]; *P* = .001), and 90° (MD, 11.8 mm [95% CI, 5.6-18.0]; *P* = .009) in comparison to the PCL-deficient (and LCL-deficient) state.

### PTT in Internal Rotation (Force Controlled)

**Figure 7.**
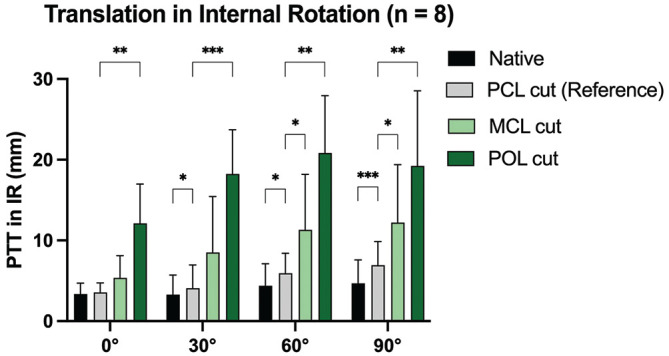
Posterior tibial translation after cutting the posterior cruciate ligament (PCL) and peripheral structures during the posterior drawer test in internal rotation. MCL, medial collateral ligament; POL, posterior oblique ligament. *P < .05; **P < .01; ***P < .001.

When performing the posterior drawer test in internal rotation, cutting the PCL led to a significant increase in PTT at 30° (MD, 0.8 mm [95% CI, 0.2-1.4]; *P* = .01), 60° (MD, 1.6 mm [95% CI, 0.3-2.8]; *P* = .002), and 90° (MD, 2.2 mm [95% CI, 1.3-3.2]; *P* = .0009) in comparison to the native state (Figure 7). Cutting the MCL significantly increased PTT in internal rotation at 60° (MD, 5.4 mm [95% CI, 0.4-10.4]; *P* = .03) and 90° (MD, 5.3 mm [95% CI, 0.4-10.1]; *P* = .03) in comparison to the PCL-deficient state. Additional cutting of the POL led to a significant increase in PTT at 0° (MD, 8.6 mm [95% CI, 4.2-12.9]; *P* = .002), 30° (MD, 14.1 mm [95% CI, 9.3-19.0]; *P* = .0002), 60° (MD, 14.9 mm [95% CI, 8.2-21.6]; *P* = .001), and 90° (MD, 12.3 mm [95% CI, 4.7-20.2]; *P* = .007) of flexion.

## Discussion

The most important finding of this study was that the posteromedial and posterolateral structures were important restraints to PTT with the knee in internal and external rotation. This effect was so pronounced that the posterolateral structures became the primary restraints to PTT in external rotation (LCL at 0° of flexion, PT/PFL at 30°-90° of flexion), which resulted in an increase in PTT of up to 12.6 mm. The posteromedial structures became the primary restraints to PTT in internal rotation at 0° (POL) and 30° (MCL). Insufficiency of the posteromedial structures led to an increase in PTT in internal rotation of up to 14.9 mm, adding to the instability caused by cutting the PCL. In neutral rotation, the peripheral structures showed only a minor contribution. Here, the PCL was the primary restraint to PTT at all flexion angles. Nevertheless, posterolateral or posteromedial insufficiency led to increased additional instability of up to 7.6 mm (at 90° of flexion) after cutting the PCL, which is congruent to previous studies.^50^

The influence of rotation on restricting PTT has previously been investigated in multiple biomechanical studies. It was found that both internal and external rotation decreased PTT in comparison to neutral rotation. This is in accordance with the results of the present study.^3,28,39^ Regarding the posteromedial structures, a previous biomechanical study, utilizing a materials testing machine to investigate the influence of medial structures on restricting PTT during the posterior drawer test in neutral and internal rotation (both at 90° of flexion), showed that internally rotating the PCL-deficient knee decreased PTT from 14.1 mm to 4.1 mm.^38^ Sectioning the posteromedial capsule first increased PTT in internal rotation of up to 4.52 mm. Sectioning the MCL further increased PTT in internal rotation to 20.25 mm. This is comparable with the results of the present study, which showed that complete insufficiency of the posteromedial complex led to a PTT of 19.2 ± 9.3 mm at 90° of flexion (12.3-mm addition to the instability caused by cutting the PCL). Another biomechanical study found the posteromedial capsule to resist 42% of PTT when the knee was in full extension and internally rotated.^39^ These findings are comparable with the results of the present study, which showed the POL to be the major restraint to PTT in internal rotation in extension (24.2% ± 14.1% contribution). The difference in absolute values of the contribution might be explained by the differences in test setups and differences in applied forces (150 vs 89 N PTT).

Regarding the posterolateral structures, numerous biomechanical studies revealed the LCL and popliteus complex to influence PTT both in the PCL-intact and PCL-deficient states.^6,9,11^ A biomechanical study on human cadaveric knee joints found that cutting the PCL allowed PTT of up to 10 mm. This finding is comparable with the results of the present study (12.6 ± 3.6 mm PTT in neutral rotation at 90° of flexion).^32^ An additional cut of the posterolateral structures (LCL + PT) led to a significant increase in PTT to a mean of 17.6 mm. This finding is also comparable with the present study’s findings (20.3 ± 10.1 mm PTT for combined PCL and posterolateral insufficiency in neutral rotation at 90° of flexion). Although it has been previously stated that insufficiency of the posterolateral structures increases PTT in external rotation,^15,46^ the exact structures responsible for this phenomenon were unclear. The present study found that the LCL was the primary restraint to PTT in external rotation at 0°, while at all other flexion angles, it was the PT/PFL. Cutting the LCL, however, led only to a small additional increase in PTT in external rotation (up to 1.4 mm) at 0° and 30° of flexion. Cutting the PT/PFL then led to a big increase in PTT in external rotation of up to 12.6 mm, indicating that combined insufficiency has to be present to result in large clinical instability.

The present study is of clinical relevance. It has been shown that the peripheral structures of the knee are frequently injured in conjunction with the PCL. A study on 494 patients with PCL injuries revealed that 53% had combined posterior abnormalities, leading to complex instability.^43^ In these combined injuries, the posterolateral complex was most commonly injured (41.7%), followed by the MCL (13.6%).^42^ However, in both studies, only the MCL, but not the POL, or posteromedial capsule was assessed.^42,43^ This absence of investigations including the POL indicates that posteromedial injuries are underrecognized.^24,42^ Indeed, injuries to the peripheral structures are frequently missed, especially in chronic cases, because magnetic resonance imaging is frequently not able to reveal torn structures.^35,44^ This oversight poses a problem, as posteromedial or posterolateral injuries were shown to increase forces acting on the cruciate ligaments.^12,22,23,25^ Therefore, missed additional posteromedial/posterolateral injuries at the time of PCL reconstruction may have detrimental effects on postoperative outcomes and furthermore might lead to increased graft ruptures.^10,33^ Thus, a thorough clinical investigation to diagnose combined posterior instability is of high importance. The results of the present study suggest that in patients with a PCL tear, but intact peripheral structures, PTT during the posterior drawer test in either internal or external rotation decreases significantly. Conversely, increased PTT in internal or external rotation indicates an injury to either the posteromedial or posterolateral complex. Depending on the flexion angle of the knee, different structures may act to restrict PTT in rotation. For example, increased PTT in internal rotation could point to an injury of the MCL or POL. It may therefore be advisable to perform the posterior drawer test in neutral position and rotation as well as at different flexion angles. This translation into clinical practice could be investigated in future studies.

A strength of this study is that both force- and motion-controlled protocols were applied to determine the role of the peripheral structures in restricting PTT in different rotations. In motion-controlled testing, the native knee kinematics was replicated in every step. By using the principle of superposition, the contribution of each cut structure in restricting PTT, irrespective of the cutting order, can be calculated.^41,54^ The requirements of superposition were all met in this study.^41,54^ In force-controlled testing, the knee was manipulated to its limits by given forces, which allowed the identification of secondary restraints (eg, posterolateral structures in neutral rotation) after the primary restraints (eg, PCL in neutral rotation) were cut. The instability produced by a cutting step during force-controlled testing is dependent on the previous cut, and the last structure to be cut typically leads to a major increase in laxity.^18^ This explains why, for example, during force-controlled testing, cutting the POL led to relevant additional instability at 60° and 90° of flexion (PTT in external rotation), while during motion-controlled testing, the contribution of the POL at these flexion angles was negligible.

This study has several limitations to consider when interpreting the results. As with most cadaveric biomechanical studies, knee specimens of an older age were used for testing. The PCL was always the first structure to be cut after determining the native knee kinematics. Therefore, the results of the present study are only relevant for PCL-deficient knees. Especially in full extension, the contribution of the PCL and all peripheral structures was <50%, indicating further structures that significantly influence PTT. Recent biomechanical studies indicated that the posterior capsular structures, including the oblique popliteal ligament, might influence hyperextension and possibly PTT near extension.^34^ No analysis of the posterolateral structures in internal rotation or the posteromedial structures in external rotation was performed during force-controlled testing, as they were found to be irrelevant in these movements during motion-controlled testing.

## Conclusion

The peripheral ligamentous structures of the knee acted as secondary restraints to PTT in neutral rotation and became primary stabilizers in internal or external rotation, depending on the flexion angle.

## Supplemental Material

sj-pdf-1-ajs-10.1177_03635465251317209 – Supplemental material for Restriction of Posterior Tibial Translation During the Posterior Drawer Test in Internal or External Rotation Is Dependent on Peripheral Stabilizers of the Knee: A Biomechanical Robotic Investigation Supplemental material, sj-pdf-1-ajs-10.1177_03635465251317209 for Restriction of Posterior Tibial Translation During the Posterior Drawer Test in Internal or External Rotation Is Dependent on Peripheral Stabilizers of the Knee: A Biomechanical Robotic Investigation by Adrian Deichsel, Christian Peez, Wenke Liu, Michael J. Raschke, Alina Albert, Thorben Briese, Elmar Herbst and Christoph Kittl in The American Journal of Sports Medicine
