# Supplementary material for: Restriction of Posterior Tibial Translation During the Posterior Drawer Test in Internal or External Rotation Is Dependent on Peripheral Stabilizers of the Knee: A Biomechanical Robotic Investigation
Source: Am J Sports Med. 2025 Feb 19;53(5):1077–84. doi: 10.1177/03635465251317209 (PMC11951389; doi:10.1177/03635465251317209)
Supplement: sj-pdf-1-ajs-10.1177_03635465251317209 – Supplemental material for Restriction of Posterior Tibial Translation During the Posterior Drawer Test in Internal or External Rotation Is Dependent on Peripheral Stabilizers of the Knee: A Biomechanical Robotic Investigation [file sj-pdf-1-ajs-10.1177_03635465251317209.pdf]

Restriction of a Posterior Drawer Test in Internal or External Rotation is Dependent on Peripheral Stabilizers of the Knee – A Biomechanical Robotic Investigation

Table A1 *Contributors to Restriction of Posterior Tibial Translation in Neutral. External. and Internal Rotation. Significant restraints are marked bold. The primary restraints are marked red.*

	PCL		LCL		PT/PFL		MCL		POL	
	Mean ± SD		Mean ± SD		Mean ± SD		Mean ± SD		Mean ± SD	
Neutral Rotation										
0°	**24.4**	**± 12.7**	6.6	± 6.5	3.6	± 4.2	5.7	± 2.5	4.2	± 2.2
30°	**52.3**	**± 26.6**	1.7	± 2.3	2.9	± 3.8	5	± 6.7	3.3	± 4.5
60°	**61.2**	**± 23.5**	0.7	± 0.7	1.4	± 1.7	6.8	± 6	1.6	± 2.1
90°	**59**	**± 16.3**	0.5	± 0.6	1.5	± 1.9	3.9	± 2.6	1.4	± 1.4
External Rotation										
0°	10.5	± 6.2	**24.7**	**± 10.5**	**16.8**	**± 17.8**	3.9	± 8.3	0.3	± 0.5
30°	**15.7**	**± 13.7**	**20.9**	**± 18.1**	**56.4**	**± 22.1**	0.1	± 0.2	1	± 1.6
60°	**32.1**	**± 22.3**	7.9	± 13	**65.2**	**± 16.7**	0	± 0	0.7	± 1
90°	**37.6**	**± 22.9**	2.9	± 5.2	**58.7**	**± 13.0**	1.4	± 3.5	0.8	± 0.7
Internal Rotation										
0°	**13.2**	**± 7.8**	0.1	± 0.1	0.1	± 0.2	10.6	± 3.2	**24.2**	**± 14.1**
30°	**13.5**	**± 14.1**	0.2	± 0.3	0.3	± 0.4	**33.6**	**± 11.4**	**23.2**	**± 17.5**
60°	**46.2**	**± 36.1**	0	± 0	0.5	± 1.1	**31.9**	**± 15.7**	5.2	± 5.5
90°	**57.8**	**± 28.6**	0	± 0	0.3	± 0.5	**17.8**	**± 14.2**	2	± 2.4
